# Management of Cutaneous Adverse Drug Reactions via Personalized Ayurvedic Interventions: A Case Report

**DOI:** 10.7759/cureus.71344

**Published:** 2024-10-13

**Authors:** Niranjan Ram, Meena S Deogade, Tanuja M Nesari

**Affiliations:** 1 Dravyaguna (Traditional Pharmacology and Indian Materia Medica), All India Institute of Ayurveda, New Delhi, IND; 2 Ayurveda Pharmacology, All India Institute of Ayurveda, New Delhi, IND; 3 Ayurveda Pharmacology, All India institute of Ayurveda, New Delhi, IND

**Keywords:** adverse drug reactions (adr), ayurveda, case report, complementary and alternative medicine(cam), cutaneous adverse drug reactions (cadrs), dermatitis, drug allergy, herbal medicine, khadirarishta, skin disorders

## Abstract

Cutaneous adverse drug reactions (CADRs), challenging medication reactions, significantly impact patient well-being and management. This case report presents the successful management of CADR (Aushadha anurjata janya twak vikara) in Ayurveda. This study uniquely demonstrates the efficacy and long-term success of personalized Ayurvedic interventions in managing treatment-resistant drug-induced dermatitis (ADR) exacerbated by conventional treatments, contributing insights into integrative approaches for drug-induced skin reactions and their potential for sustained remission. The condition involved widespread erythematous rashes, severe burning sensations, and skin scaling, which worsened after conventional medication use. Clinical findings revealed extensive skin involvement, characterized by erythema (redness) and epidermal barrier dysfunction (skin disruption). The patient was diagnosed with inflammatory, drug-induced skin manifestations, corresponding to Pitta-predominant characteristics in Ayurveda, and was prescribed personalized treatments, including herbal and herbo-mineral formulations such as Shirisharishta, Khadirarishta, Panchanimba churna, Rasamanikya, Gandhaka Rasayana, and Tankana Bhasma, and topical applications of medicated oils and decoctions. Out of a total of four visits, treatment led to significant improvement within 2 weeks, near-complete resolution after 3 weeks, and the patient was completely asymptomatic at the fourth visit. He was followed for the next year. One-year follow-up, including biweekly visits (first month), monthly telephone updates (next 5 months), and bimonthly updates (final 6 months), revealed no symptom relapse. This case highlights two key takeaways - the potential of personalized Ayurvedic approaches for managing treatment-resistant drug-induced dermatitis and achieving long-term remission through integrative strategies in complex dermatological cases. This case underscores the need for further research into the role of traditional medicine in managing challenging skin disorders and maintaining prolonged symptom-free periods.

## Introduction

Adverse drug reactions (ADRs) are defined as “a response to a drug that is noxious and unintended, and which occurs in doses normally used for the treatment, prophylaxis, or diagnosis of disease, or the modification of physiological function” [1]. ADRs encompass all types of adverse reactions to drugs, affecting any part of the body. Among all types of ADRs, when the unintended and harmful reaction to a systemic drug is limited to only the skin, it is termed CADR or toxidermia. These reactions range from mild erythematous skin lesions to severe forms of drug-induced dermatitis, including bullous reactions (Stevens Jhonson syndrome (SJS) and toxic epidermal necrolysis (TEN)/Lyell syndrome), acute generalized hyperemic pustulosis (AGEP), and drug reactions with eosinophilia and systemic symptoms (DRESS) [2]. CADR is frequent, but a small fraction (2%) of adverse reactions is classified as severe, and very few are fatal. Only 2-3% of all hospitalized patients suffer from CADR. Fortunately, most drug eruptions are self-limited, mild, and resolved with the discontinuation of the offending pharmacological agent. Severe and potentially life-threatening events such as SJS and TEN are rare and affect approximately 1 or 2 out of one million people [3-4]. They are characterized by a heterogeneous field of clinical presentations, sometimes with exanthematous (maculopapular) drug eruption and sometimes with no obvious features suggesting drug causality. It can significantly impact a patient's quality of life and pose challenges in management, especially when the causative agent is part of the necessary treatment for another condition. In Ayurveda, this condition can be correlated with Aushadha anurjata janya twak vikara, which refers to skin disorders caused by incompatible or improperly administered medicines. This concept of Aushadha anurjata (allergic to certain groups of medications) can be categorized under the broad umbrella of Asatmya (incompatibility) in Ayurveda. A substance that is not favorable to the human body is known as Asatmya, and it becomes responsible for vitiating and provoking the internal milieu of the organism [5].

Ayurveda, the ancient system of medicine, focuses on a holistic approach to treating skin disorders. It considers not only the local manifestations but also the systemic imbalances that may contribute to the condition. The Ayurvedic approach to skin disorders is based on the concept of the harmony between three Doshas (regulatory functional factors of the body) (i.e., Vata (dosha responsible for movement and cognition), Pitta (dosha responsible for regulating body temperature and metabolic activities) and Kapha (dosha responsible for regulating body fluids and keeping the body constituents cohesive)), rhythmic transformation of seven Dhatus (major structural components of the body), proper elimination of Mala (Sharira mala and dhatu mala) (waste products) from the human body and their role in maintaining skin health. CADR, from an Ayurvedic perspective, is often seen as a result of aggravated Pitta dosha, with possible involvement of Vata and Kapha depending on the specific manifestations [6]. This case report presents the Ayurvedic management of drug-induced dermatitis that developed following the use of conventional medications for skin conditions.

## Case presentation

Patient information

**Figure 1:**
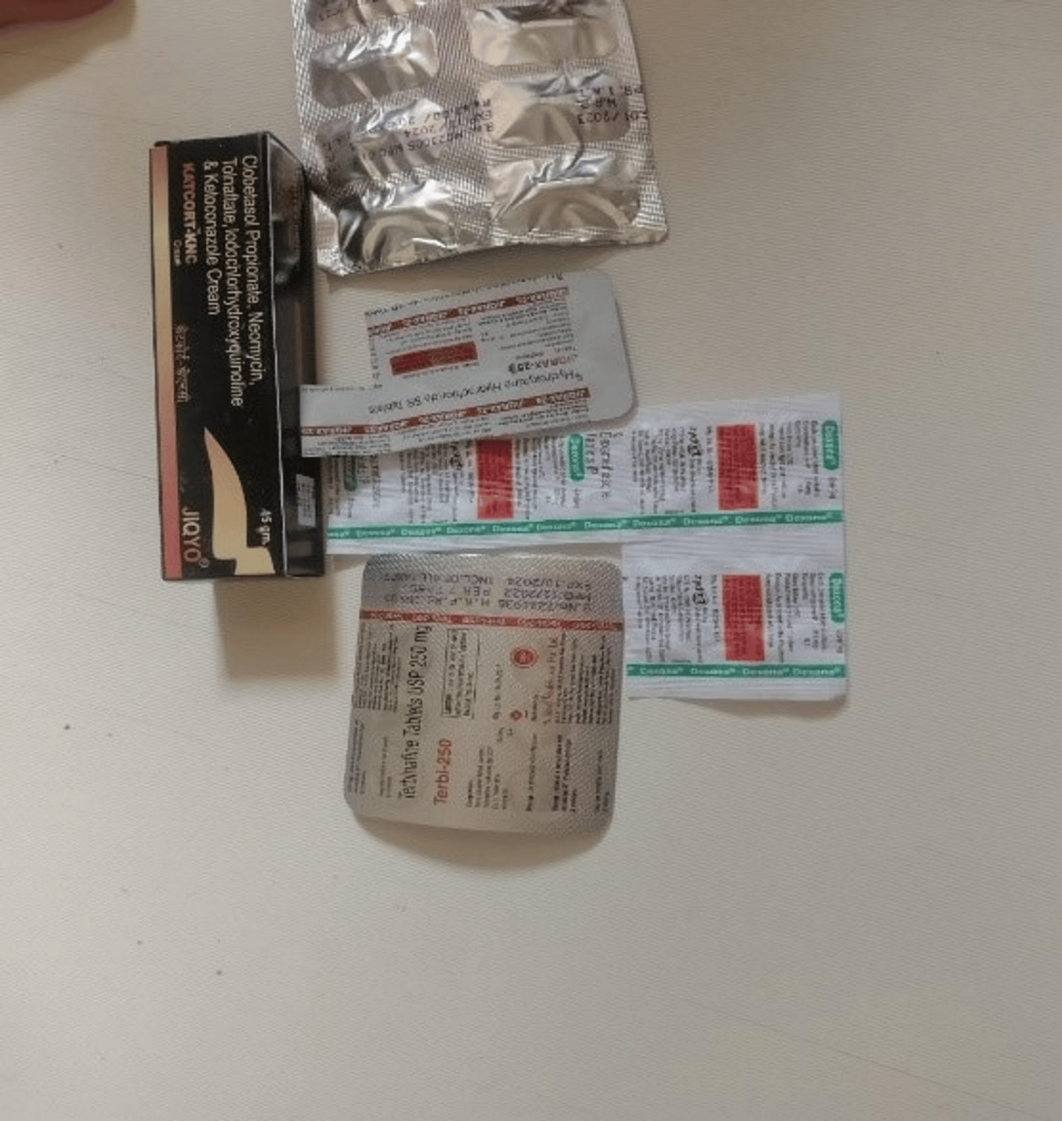
Conventional medicines used (dated: 12/08/2023)

A 32-year-old male patient, employed as a glazier (working in the glass industry, basically for cutting, installing, and removing glass), presented to the skin OPD of a tertiary Ayurvedic hospital with severe skin manifestations. The patient had initially developed a skin problem associated with itching, redness, and urticarial patches for which he had consulted local practitioners. He had no significant past medical or psychological history and no known allergies prior to this incident. The patient reported no family history of similar skin conditions or allergies and no relevant genetic involvement. No significant past interventions were noted as a consequence of which this urgency had developed. However, related to these skin manifestations, he took the following conventional medications, after which the condition worsened: terbinafine (antifungal), dexamethasone (corticosteroid), hydroxyzine (antihistamine), and pantoprazole (proton pump inhibitor) and a topical combination of clobetasole (corticosteroid), neomycin (antibiotic), tolnaftate (antifungal), iodochlorhydroxyquinoline (antimicrobial and antifungal), and ketoconazole (antifungal). Unfortunately, he had no prescriptions, most likely taken by the advice of local practitioners or as an over-the-counter (OTC) product (Figure 1).

The patient’s occupation as a glazier is noteworthy, as it involves continuous exposure to glass dust, cleaning agents, and adhesives or sealants, which could contribute to skin irritation. His lifestyle included a mixed diet, moderate physical activity related to his work, and no reported substance use. The sleep patterns of the patient were disturbed during this period because of the itching caused by these skin manifestations.

Clinical findings

**Figure 2:**
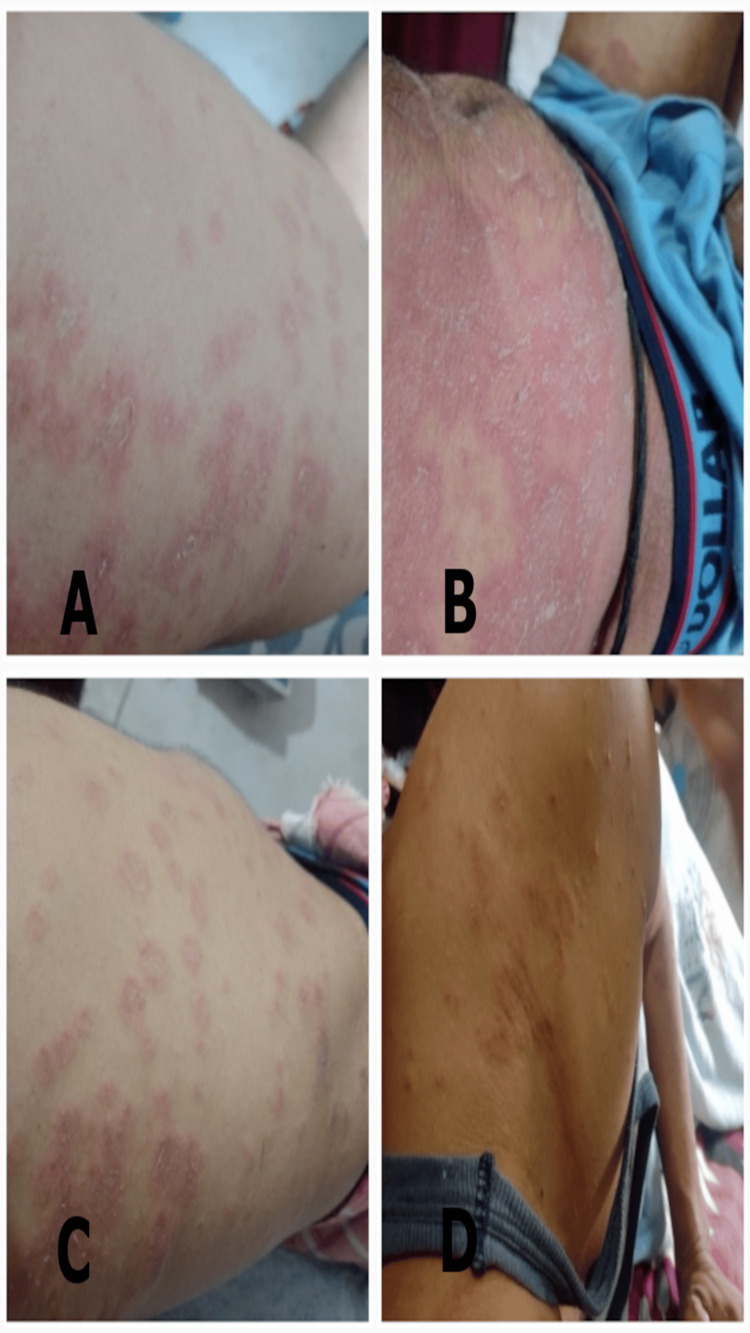
The extent of erythematous patches with itching and burning at first visit (12/08/2023) A) in the left thigh B) in abdomen C) in right flank and back D) in the left shoulder area.

**Figure 3:**
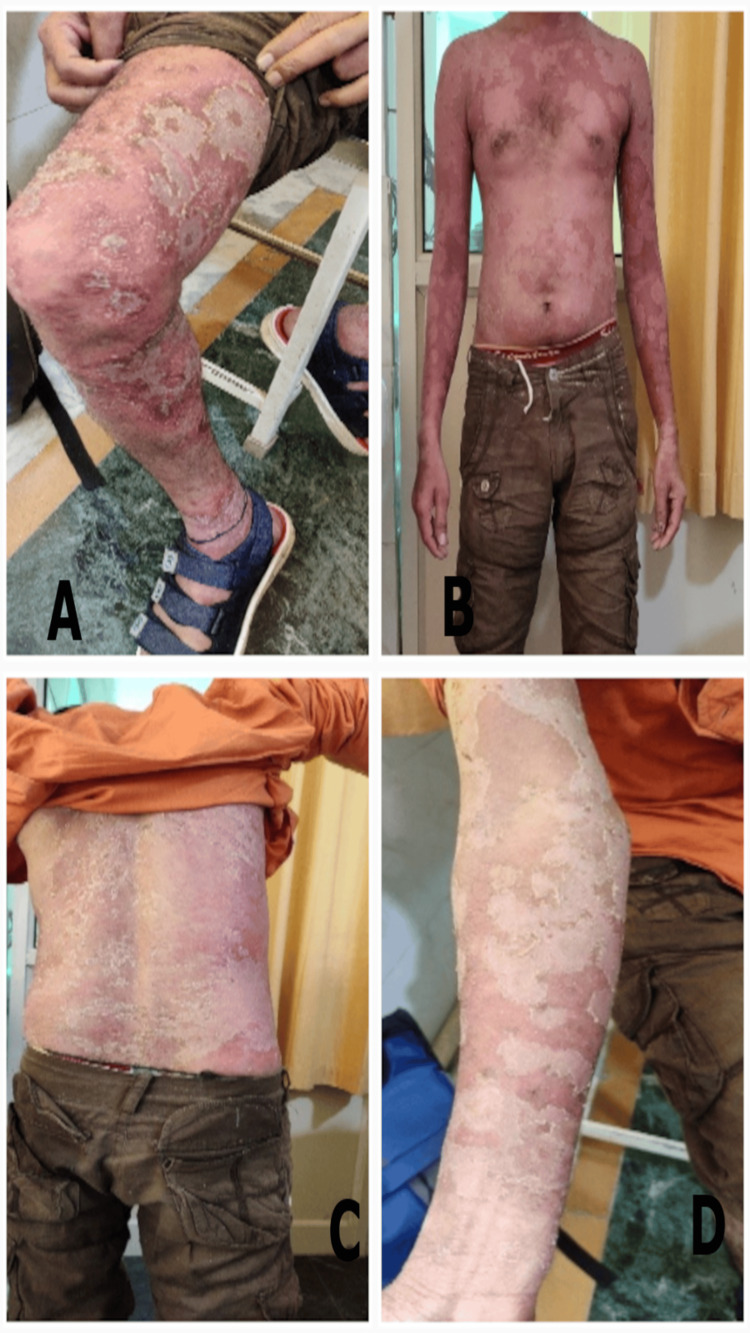
The extent of erythematous patches with itching, burning, and scaling at the second visit (16/08/2023) A) in the right thigh B) in abdomen and chest C) in back D) in right forearm.

**Figure 4:**
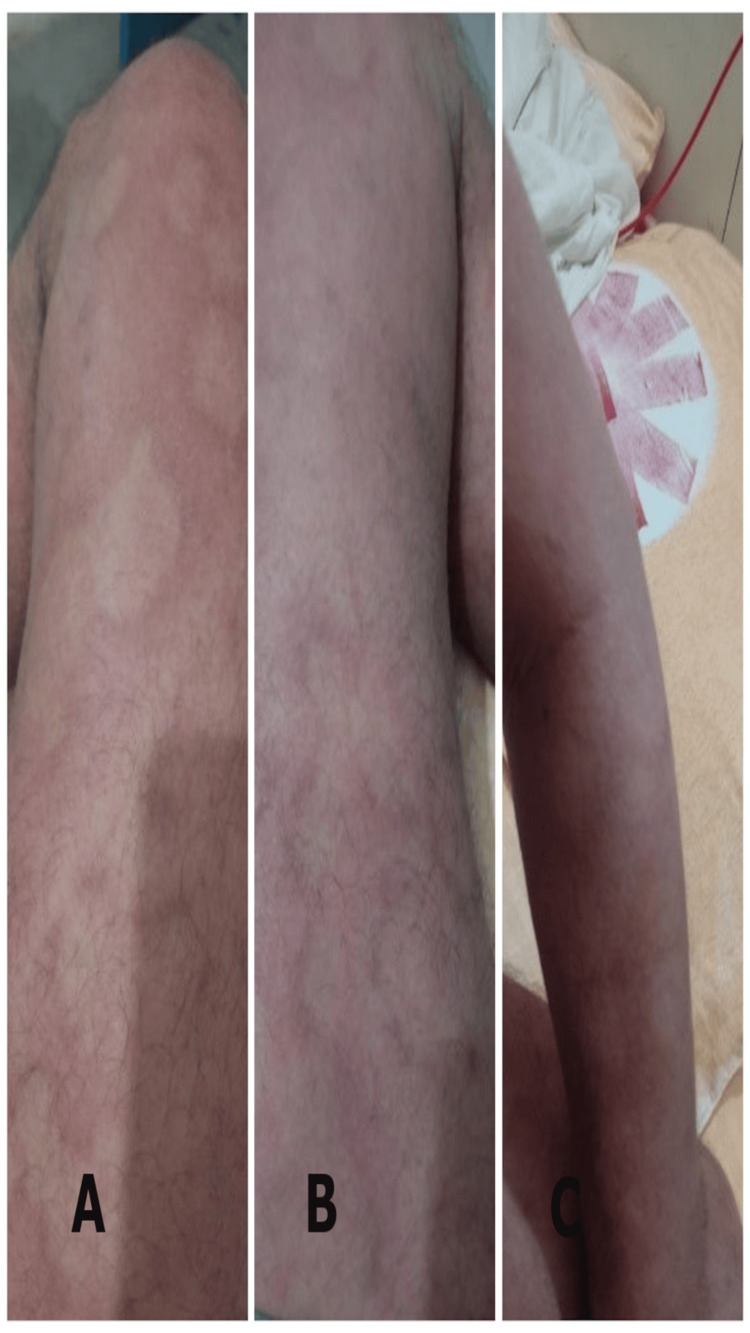
Partial remission of erythematous patches (dated 22/08/2023) A) in the right thigh, B) in left thigh, and C) in left hand.

On initial examination, the patient presented the following physical findings: widespread erythematous rashes covering large areas of the body, particularly prominent on the trunk and extremities; mild itching and a burning sensation, causing significant discomfort and sleep disturbance; noticeable scaling of the skin, indicating epidermal involvement; and increased local skin temperature and mild edema in the affected areas (i.e., trunk and extremities) (Figures 2-4).

All vital signs were within normal limits, i.e., body temperature = 99°F, pulse rate = 80/min, respiration rate = 20/min, and blood pressure = 126/80 mmHg. The patient reported that the itching intensified and that the rash spread more rapidly after initiating conventional medications. He also experienced a severe burning sensation and increased skin sensitivity, which were not present before starting the treatment.

Astavidha Pareeksha (Eightfold examination)

Nadi (pulse) was Pitta-Kapha dominant in character; Mala pravrutti (elimination of excreta) was Asamyak (irregular); Mutra (urine) was normal in color; Jihwa (tongue) was pale, thick white coating at the back and slightly more reddish at the sides; Shabda (voice) was medium pitch but somewhat weak; Sparsha (tactile examination) was warm and rough at the involved areas of trunk and peripheries; Drik (eye) was slightly red and irritated; and Akruti (body stature) was moderate.

Timeline

A detailed timeline of events is illustrated in Table 1.

**Table 1:** Timeline of detailed events Naranjo scale: adverse drug reaction probability scale; DLQI: Dermatology Life Quality Index

Date	Observation and remarks
Day 0 (02/08/2023)	Initial onset of skin problems with mild itching and burning
Day 10 (12/08/2023)	First visit; diagnosed as CADR, Naranjo scale and DLQI assessment were done, and initial Ayurvedic treatment prescribed.
Day 14 (16/08/2023)	Second visit; symptoms worsened, the Naranjo scale and DLQI reassessed, treatment plan modified.
Day 20 (22/08/2023)	Third visit; significant improvement noted, Naranjo scale and DLQI reassessed, treatment adjusted.
Day 27 (29/08/2023)	Fourth visit; almost complete resolution of symptoms, Naranjo scale and DLQI finally assessed and no medications advised.
1^st^ Month	Follow-up at a 15-day gap, no reversal of symptoms, and no medications advised.
2^nd^ – 5^th^ Month	Follow-up with telephonic conversation at 30 days interval, no symptoms, and no medications.
6^th^ – 12^th^ month (Completed on 30/08/2024)	Follow-up with telephonic conversation at 60 days interval, no symptoms, and no medications.

Diagnostic assessment

On the basis of the patient’s history and clinical presentation, a diagnosis of CADR (Aushadha anurjata janya twak vikara) was made. The aggravation of symptoms following conventional medication use, coupled with the characteristic skin manifestations, strongly supported this diagnosis. To assess the severity of the problem, a complete blood count (CBC), liver function test (LFT), renal function test (RFT), inflammatory marker-erythrocyte sedimentation rate (ESR), C-reactive protein (CRP), immunoglobulin-E (IgE), skin biopsy, and allergy tests were advised. Owing to monetary issues with the patient, only the CBC, LFT, RFT, and inflammatory markers (ESRs) were checked. Elevated white blood cell (WBC) (i.e., 15500/cu mm), neutrophil (92%), and eosinophil (9%) counts) and increased ESR (63 mm/1 h) confirmed the diagnosis of drug allergy. Along with pathological investigations, two scales were used to assess the severity and impact of the ADR: the Adverse Drug Reaction Probability Scale (Naranjo scale) [7] and the Dermatology Life Quality Index (DLQI) [8-9]. The initial scores at the first visit were as follows: Naranjo scale - 10 (indicating a definite ADR) and DLQI - 17 (within the 11-20 range, indicating a moderate to severe impact on the patient’s quality of life). While the Naranjo scale helped confirm the definite causality of the drug in the adverse reaction, the DLQI provided insight into the severity of the condition and its impact on the patient’s daily functioning. A score of 10 on the Naranjo scale typically indicates a definite relationship between the drug and the adverse event. This score was based on several key factors observed in our case, including, i) The adverse event appeared shortly after the initiation of the medications, particularly the oral terbinafine and dexamethasone, which are known to have potential dermatological side effects; ii) The patient’s symptoms worsened after re-exposure to the same drug regimen. This was most noticeable after the use of the topical combination containing clobetasol, neomycin, and ketoconazole; iii) Through clinical examination, we ruled out other potential causes for the adverse reaction. While pantoprazole, hydroxyzine, and iodochlorhydroxyquinoline could be involved, their side-effect profiles are less likely to cause the specific symptoms observed in this case; iv) The known side effects of the medications used, particularly terbinafine (which is associated with dermatologic reactions) and dexamethasone (which can cause skin thinning or delayed wound-healing), support the likelihood diagnosis.

However, in this particular case, the Naranjo scale was used during each follow-up visit to closely monitor the progression of the patient's symptoms and the potential impact of ongoing treatment. Given that the patient’s response to therapy evolved over time and new interventions were introduced, it was important to repeatedly assess the likelihood of an ADR throughout the course of treatment.

From an Ayurvedic perspective, the condition was assessed as a Pitta-predominant skin manifestation with secondary involvement of Vata, Kapha Doshas and consideration of either Dushi visha (artificial poison) or Agantuja visha (exogenous poison) for the conventional drugs causing adverse skin manifestations. The features suggesting Pitta involvement included erythema, a burning sensation, and rapid progression of the rash. Vata involvement was indicated by intense itching, dryness, and scaling of the skin, whereas Kapha involvement was suggested by mild edema at the affected parts, itching, and thickening of the affected skin areas. This Pitta vitiation, triggered by the Dushi visha or Agantuja visha (in this case, the conventional medications), provides the basis for the Ayurvedic diagnosis and subsequent treatment approach. The diagnosis also considered the concept of Satmya (homologation) in Ayurveda, which suggests that the prescribed conventional medications were incompatible with the patient's constitution, leading to an adverse reaction manifesting as severe dermatitis.

Therapeutic intervention

The foremost therapeutic advice in this case of CADR was to stop all conventional medications due to which the situation arose and became complicated. Simultaneously, to assess the status of the disease and strength of the patient, the following therapeutic interventions were prescribed and modified over the course of four visits to address the changing needs of the patient and the progress of the condition. The details of the therapeutic interventions are summarized below in Table 2.

**Table 2:** Details of therapeutic interventions administered

Date	Medications	Dose, frequency	Rationale
12/08/2023 (First visit)	Panchanimba churna (3 g) + Rasamanikya (60 mg) + Gandhaka Rasayana (250 mg) with honey	Combined dose as described, twice a day after food	Aimed to pacify the aggravated Pitta dosha. Reducing inflammation and initiating the detoxification process
	Khadirarishta with an equal amount of water	15 ml, twice a day after food	
	Panchatikta ghrita Guggulu with lukewarm water	5 g on an empty stomach	
	Panchavalkala Kwath + Tankana Bhasma local apply	Three times a day	
	Eranda Taila in warm milk	10 ml at bed time	
16/08/2023 (Second visit)	Panchanimba churna (3 g) + Rasamanikya (60 mg) + Gandhaka Rasayana (250 mg) with honey	Combined dose as described, twice a day after food	The washout period of the conventional medicines was not over but the patient started Ayurvedic medications, so initially, symptoms aggravated (maybe any interaction between the two). Incompatible with Panchatikta ghrita guggulu and Eranda taila, so modified. Sanjeevani vati was added to combat fever.
	Khadirarishta + Shirisharishta with equal amount of water	10 ml each, twice a day after food	
	Panchavalkala Kwath + Panchatikta Kwath + Tankana Bhasma local apply	Three times a day	
	Jatyadi taila + Yashtimadhu taila for topical applications after washing and complete drying	Three times a day	
	Avipattikara churna	6 g at night before dinner	
	Sanjeevani vati	250 mg twice a day after food as the fever continued	
22/08/2023 (Third visit)	Panchanimba churna (3 g) + Rasamanikya (60 mg) + Gandhaka Rasayana (250 mg) with honey	Combined dose as described, twice a day after food	Significant improvements in symptoms were noted. Nalapamaradi taila was added due to its specific action and varnya activity (~restoring normal complexion) Haridra Khanda was added to support overall immunity and skin health.
	Khadirarishta + Shirisharishta with equal amount of water	10 ml each, twice a day after food	
	Panchavalkala Kwath + Panchatikta Kwath + Tankana Bhasma local apply	Three times a day	
	Jatyadi taila + Yashtimadhu taila for topical applications after washing and complete drying	Three times a day	
	Nalapamaradi taila for topical application – 1 hour before bath	Once a day	
	Haridra Khanda	5 g twice a day with lukewarm milk	
29/08/2023 (Fourth visit)	No medications were advised	-----------	Patient is asymptomatic

Along with these medications, the patient was advised to follow the dietary regimen as customized for him. He was advised to avoid hot, sour, salty, spicy, and fermented food items as they may worsen the symptoms.

Follow-up and outcomes

**Figure 5:**
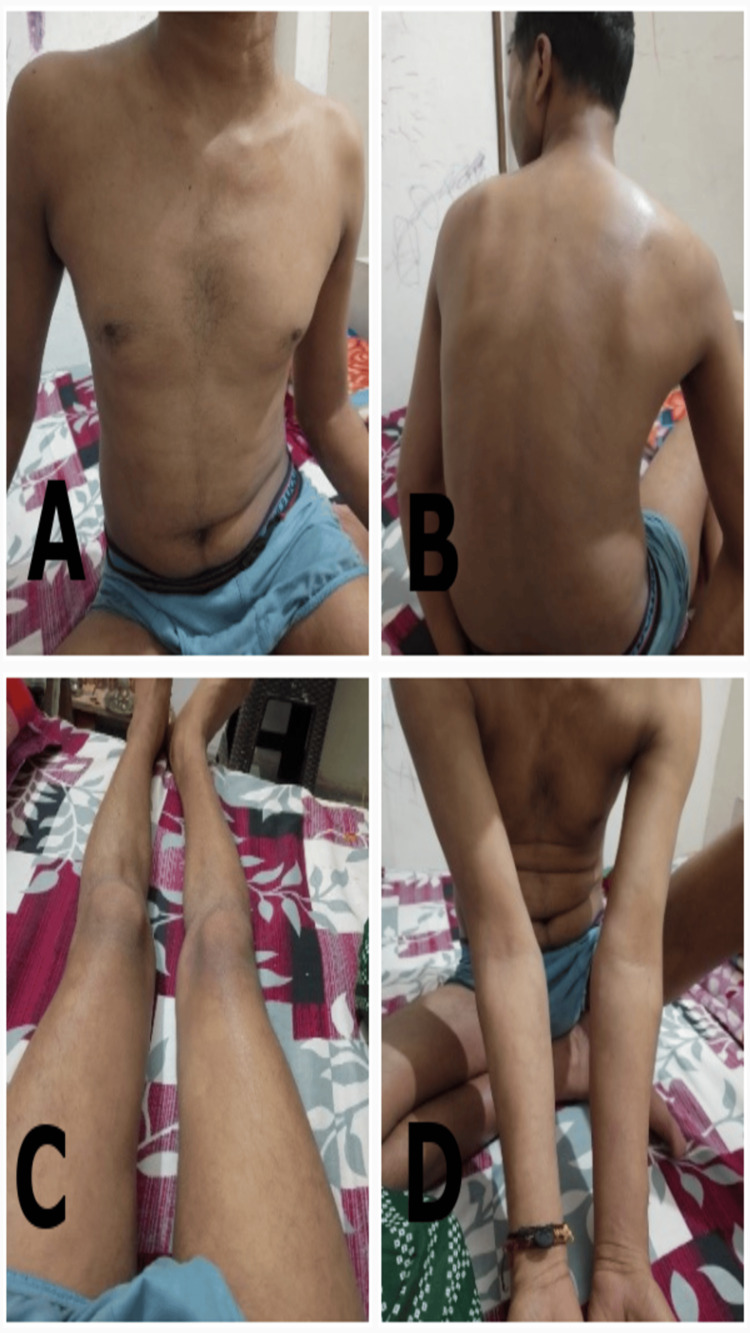
Complete remission of erythematous patches and restoration of normal complex (dated: 29/08/2023) A) in the abdomen and chest B) in back C) in lower limbs, and D) in upper limbs.

One week after the third visit’s treatment plan, i.e., on 29/08/2023, the patient reported almost complete resolution of symptoms. The skin appeared clear, with a significant reduction in erythema, scaling, and itching (Figures 5A-5d).

The patient expressed feeling healthy and happy, with improved sleep and a return to normal daily activities. The objective improvements are the resolution of erythema and rash, absence of scaling and edema, normal skin temperature, and no reports of itching or burning sensations. All of the pathological outcomes and the Naranjo scale and DLQI scores were normal and are presented in Table 3.

**Table 3:** Assessment of pathological parameters before and after treatment

Parameters	Before treatment	After treatment
Hematological parameters		
Total leucocyte count (/cu mm)	15,500	7800
Neutrophil count	92	53
Eosinophil	9	5
ESR (mm in 1^st^ hour)	63	21

The assessment and interpretation of the extent of adverse drug reactions in the Naranjo scale and DLQI scores are illustrated in Tables 4-5.

**Table 4:** Assessment of adverse drug reactions in Naranjo scale and DLQI score DLQI: Dermatology Life Quality Index

Scale	Day 0	Visit 1	Visit 2	Visit 3	Visit 4
Naranjo Scale (Adverse Drug Reaction Probability)	7	10	12	6	1
DLQI (Dermatology Life Quality Index) Score	6	17	25	5	1

**Table 5:** Interpretation of Naranjo scale and DLQI (Dermatology Life Quality Index) scores from the standard reference range

Interpretation of results in Naranjo scale		Interpretation of results in DLQI score	
Score	ADR probability	Score	Effect on life
≤ 0	Doubtful	0 - 1	No effect
1 - 4	Possible	2 - 5	Small effect
5 - 8	Probable	6 - 10	Moderate effect
≥ 9	Definite	11 - 20	Very large effect
		21 - 30	Extremely large effect

The patient adhered well to the prescribed medications, compliance was confirmed by regular interaction with the patient via telephone, and no adverse effects from the Ayurvedic treatment were reported.

**Figure 6:**
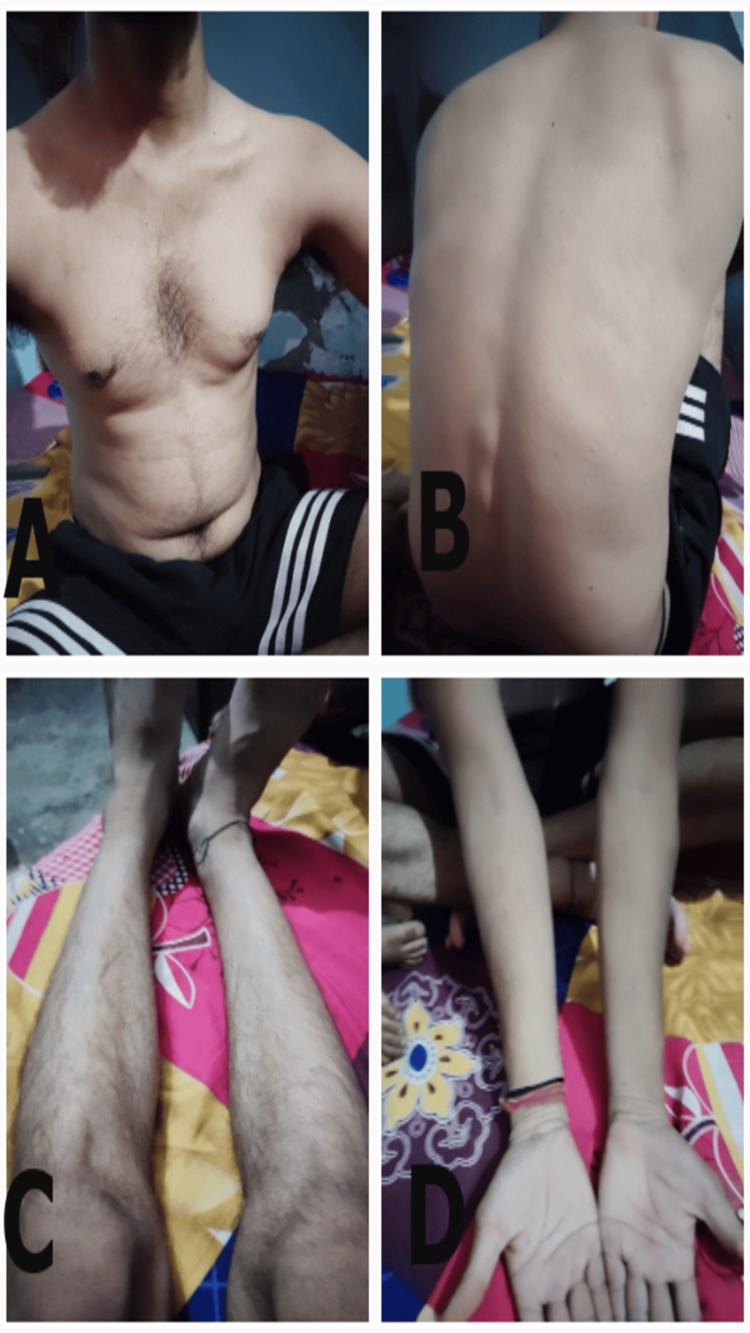
No signs of relapse and maintenance of skin health even after a year (dated: 30/08/2024) A) in the abdomen and chest B) in back C) in lower limbs, and D) in upper limbs.

Long-term follow-up was conducted for one year post-treatment. The well-being of the patients was assessed at fifteen-day intervals for the first month, once a month for the next five months, and bimonthly for the final six months over telephone interactions. Throughout this extended follow-up period, the patient remained symptom-free, with no signs of relapse (Figures 6A-6D).

The skin maintained its normal appearance and function, and the patient reported no recurrence of itching, rash, or any other symptoms associated with the initial condition.

Patient perspective

The patient expressed high satisfaction with the treatment outcomes. In his words, “I had almost lost hope after my condition worsened with the previous medications. The Ayurvedic treatment not only cleared my skin but also made me feel better overall. I appreciate the holistic approach and the absence of any noticeable side effects. It is remarkable how my skin has healed, and I am able to return to my normal life.”

Informed consent

The authors certify that patient informed consent was obtained for the purpose of publication of this case report, including the use of clinical photographs and other clinical information in the journal. He understood that his name and initials would not be published and that proper efforts would be taken to conceal his identity; however, complete anonymity cannot be assured. He was made aware of the potential benefits and risks of the Ayurvedic treatment approach, and he willingly opted for this line of management.

## Discussion

This case establishes the efficacy of Ayurvedic interventions in managing CADR. The treatment approach focused on pacifying aggravated Doshas, enhancing skin health, and addressing systemic imbalances. The key strengths in managing this case include customizing the treatment according to the patient's specific symptoms and constitution; the holistic approach addresses both local skin manifestations and systemic imbalances; multicomponent formulations provide a synergistic effect in managing the complex pathology of drug-induced dermatitis; gradual improvement, leading to long-lasting results without rebound effects; good tolerance and no reported side effects of Ayurvedic medications, in contrast with the adverse reactions experienced from conventional medications; and long-term efficacy without any relapse over one year of follow-up, although the patient returned to his occupation and continued the same over the year.

However, several limitations are also present. The dependence on telephone follow-up for a major portion of the observation period may have resulted in the absence of subtle clinical changes. However, as there was no emergency from the patient’s side and the whole family was dependent on his daily income, he was unable to attend the OPD physically. Additionally, as a single case report, the results are not generalizable across the entire spectrum of CADR. One of the limitations of this study is the absence of a few laboratory investigations, such as microbial culture and sensitivity testing, KOH study, histopathological analysis, skin biopsy, and other allergy tests due to ethical concerns and financial constraints from the patient aspect. These investigations could have provided further insight into the systemic effects of the culprit drugs, as well as the potential interactions with the Ayurvedic medications used. Despite this limitation, the diagnosis was well-supported by clinical and laboratory findings, including imaging and blood markers, which were consistent with the suspected ADR.

CADRs are a well-recognized phenomenon in modern dermatology. CADR can present with a spectrum of clinical symptoms, ranging from relatively benign maculopapular rashes to life-threatening severe cutaneous adverse drug reactions, known as SCAR. While this case represents a milder form of CADR, SCAR is a subset of CADR and includes drug-induced hypersensitivity syndrome (DIHS)/drug reactions with eosinophilia and systemic symptoms (DRESS), SJS and TEN, which are rare but fatal. The mechanism behind CADR, including the SCAR, involves T-cell-mediated immune reactions induced by medications. These medications act as foreign antigens, binding to T-cell receptors (TCRs) and initiating immune responses. Genetic factors, such as human leukocyte antigen (HLA) alleles, can predispose individuals to certain drug reactions, suggesting a complex interaction between HLA, drug, and environmental factors in the development of CADR and SCAR [10]. The most common drugs responsible for the majority of cases of CADR reported are antibiotics (beta-lactams, sulfonamides), nonsteroidal anti-inflammatory drugs (NSAIDs), antiepileptics (carbamazepine, hydantoins), and allopurinol. CADR involves a reaction to a combination of medications, including antifungals, antihistamines, and corticosteroids [2].

In Ayurveda, CADR (Aushadha anurjata janya twak vikara) is understood as a manifestation of disturbed Dosha balance, particularly Pitta dosha, which governs metabolism and transformation in the body. The condition is seen as a result of the body’s reaction to substances that are inherently incompatible (Asatmya) with the individual’s constitution. Ama (endotoxin) and Dushi visha or Agantuja visha (conventional medications used here) have also played crucial roles in skin manifestations [5]. The pathogenesis involves the following concepts: Viruddha ahara/aushadha (antagonistic food or medicine combinations). A mixture of various medications is viewed as potentially incompatible, leading to adverse reactions. Agnimandya (weakened metabolic factors): The use of multiple medications can overcome the body's metabolic capacity, leading to the formation of Ama. Dosha prakopa (aggravation of Doshas): Incompatible medications primarily aggravate Pitta, with secondary involvement of Vata and Kapha. Dhatu dushti (damage to the major structural components of the body): The Doshik imbalance in the body later involves Dhatus, primarily Rasa (primary product of the digested food), and Rakta dhatu (blood tissue), which leads to skin tissue damage, manifesting as various dermatological symptoms.

The Ayurvedic treatment approach mainly focuses on pacifying aggravated Doshas, primarily Pitta; eliminating accumulated toxins (Ama, Dushi visha, and Agantuja visha); enhancing the strength of Dhatus, particularly Rasa, which is responsible for skin health; restoring the normal functioning of Agni (metabolic factors); and providing symptomatic relief from itching, burning, and other discomforts. It must be noted here that - in spite of a holistic approach, the symptoms were aggravated in the second visit (16/08/2023). It may be due to the patient having started Ayurvedic medicines and the washout period of conventional medications was not over, probably resulting in herb-drug interactions.

The drugs used in this case report are the most frequently used classical drugs. Their potential in the efficient management of CADR is also well established by contemporary science. Khadirarishta and Shirisharishta are fermented herbal preparations that possess anti-inflammatory, antimicrobial, and immune-modulatory properties. The bark of Khadira [*Acacia catechu* (L.f.) Willd.] It is rich in tannins and flavonoids, resulting in its antioxidant, anti-inflammatory, antibacterial, and antifungal properties, which contribute to its effectiveness in treating skin disorders. It acts by reducing capillary permeability and local inflammation [11]. Shirisha [*Albizia lebbeck* (L.) Benth] has antiallergic properties, inhibiting the release of histamine and stabilizing mast cells. The fermentation process enhances the bioavailability of active compounds and increases probiotic benefits [12]. Panchanimba churna is a polyherbal formulation that is primarily composed of various useful parts (i.e., roots, stem bark, leaves, flowers, and fruit) of the famous bitter herb Neem (*Azadirachta indica* A. Juss.), has potent anti-inflammatory, antimicrobial, and blood-purifying properties. The bitter taste of these herbs stimulates the liver, enhancing detoxification processes. They also have an antipyretic effect, helping to reduce local heat manifestation in inflamed skin areas [13]. Rasamanikya is an Ayurvedic mineral preparation with arsenic as its main ingredient, and it possesses powerful detoxifying and rejuvenating properties. In carefully processed forms, arsenic compounds can modulate immune responses and have been traditionally used to treat skin diseases. It is believed to act at a deep tissue level, supporting the regeneration of healthy skin cells [14]. Gandhaka Rasayan is a sulfur-based preparation known for its effectiveness in treating skin disorders because of its antimicrobial and keratolytic properties. Sulfur has been used in dermatology for its anti-inflammatory and antipruritic effects. It also helps reduce excessive oiliness of the skin, which can be beneficial in certain types of dermatitis [15]. Panchavalkala and Panchatikta kwath are herbal decoctions that have astringent, anti-inflammatory, and wound-healing properties. Panchavalkala, which is composed of bark from five different trees, is rich in tannins that provide an astringent effect, reducing exudation and promoting healing [16]. Panchatikta kwath, made from bitter herbs such as Guduchi [*Tinospora cordifolia* (Willd.) Miers] and Kiratatikta [*Swertia chirayita* (Roxb.) Buch. -Ham. ex C.B. Clarke], has anti-inflammatory, antimicrobial, and antipyretic properties, helping to reduce infection risk and inflammation [17]. Tankana (Borax) bhasma, which, when administered along with these two decoctions, accelerates the process through its antimicrobial and pH-balancing properties. Jatyadi taila and Yashtimadhu taila are medicated oils that have wound-healing, anti-inflammatory, and skin-nourishing properties. Jatyadi taila contains herbs such as Jati (*Jasminum officinale* Linn.) that promote granulation tissue formation and epithelialization. Yashtimadhu (*Glycyrrhiza glabra* L.) has anti-inflammatory properties and can inhibit melanin production, promoting postinflammatory hyperpigmentation [18]. Nalapamaradi Taila is specifically indicated for various skin disorders and possesses anti-inflammatory, antimicrobial, and skin-rejuvenating properties. It contains herbs that nourish the skin, reduce inflammation, and promote the growth of healthy skin tissue [19]. Haridra Khanda is a formulation rich in turmeric (*Curcuma longa* L.), which is commonly prescribed for its potent anti-inflammatory, antiallergic, and antioxidant properties. The primary bioactive compound curcumin, in turmeric, has been shown to modulate various inflammatory pathways and enhance wound-healing. It also supports liver function, aiding in overall detoxification [20]. The combination of all these formulations might effectively prevent disease pathogenesis, as a result of which the patient returned to a healthy life within a span of merely 18 days.

## Conclusions

This case report suggests that Ayurvedic treatments can be effective in managing drug-induced skin reactions, particularly in cases where conventional treatments may be contraindicated or have resulted in adverse effects. The success of this treatment can be attributed to several factors, such as the holistic approach of Ayurveda, the use of multicomponent herbal formulations that provide a synergistic effect in managing this complicated case, and the flexibility of the treatment protocol, allowing for adjustments based on the patient's response and the integration of both internal medications and external applications, providing comprehensive care for the skin and the whole body. Furthermore, the sustained remission observed over a one-year follow-up period suggests that Ayurvedic treatments may offer long-lasting benefits in managing CADR. This report provides evidence supporting the integration of traditional medicine approaches in modern healthcare, particularly in the field of dermatology. However, this is a single case report, and its findings are not broadly applicable to all cases of drug-induced dermatitis. Further research, including randomized controlled clinical trials, is needed to establish the efficacy of Ayurvedic treatments for drug-induced skin reactions on a larger scale.
